# How do I develop a psychological test or questionnaire?

**DOI:** 10.3205/zma001803

**Published:** 2026-01-15

**Authors:** Marianne Giesler, Götz Fabry

**Affiliations:** 1 Freiburg i. Brsg., Germany; 2 University Freiburg, Department of Medical Psychology and Medical Sociology, Freiburg i. Brsg., Germany

**Keywords:** phases of test- and questionnaire construction, reliability, validity, generation and wording of items

## Abstract

The purpose of this *How-to article* is to provide physicians and other health professionals working in the field of medical education research with a basic understanding of the construction of tests or questionnaire measures. The construction of such measures is too complex to be described on a few pages. Therefore, this article can only enable readers to roughly evaluate such measures or to convey an idea of how these are generally constructed.

The article outlines various phases of test or questionnaire construction. It begins with the *content phase*, in which a construct is defined, if possible, by drawing on theories and models. Here, items are written, a response format is selected, the instruction is formulated, and pilot tests are conducted. In the *structural phase*, the structure of the test or questionnaire is evaluated using suitable test statistical methods and statistical parameters. In the final phase (*external phase*), additional evidence for the validity of test or questionnaire results is sought. The validation of such measures is not the last step in the construction of tests or questionnaires as it is to be considered in all phases of test or questionnaire construction. The validation of test and questionnaire measures is theoretically and methodically demanding and should never be considered complete. Strictly speaking, it should not be said that a test or questionnaire is valid, because validity is not a property of such measures. It rather is statements and conclusions based on test or questionnaire results that can be valid.

## Abstract (de)

Ziel dieses *Gewusst-wie-Artikels* ist es, Ärztinnen und Ärzte sowie anderen Gesundheitsfachkräften, die im Bereich Medizinische Ausbildungsforschung tätig sind, ein Grundverständnis der Konstruktion von Test- oder Fragebogenverfahren zu vermitteln. Die Konstruktion solcher Verfahren ist insgesamt zu komplex, um sie auf einigen wenigen Seiten zu beschreiben. Daher kann dieser Beitrag Leserinnen und Leser lediglich in die Lage versetzen, solche Verfahren grob zu bewerten, bzw. eine Vorstellung davon zu vermitteln, wie solche Verfahren im Allgemeinen konstruiert werden.

Der Beitrag skizziert verschiedene Phasen der Test- bzw. Fragebogenkonstruktion. Er beginnt mit der *inhaltlichen Phase*, in der ein Konstrukt nach Möglichkeit mit Rückgriff auf Theorien und Modelle definiert wird. Hier werden Items formuliert, ein Antwortformat ausgewählt, die Instruktion formuliert und Vortests durchgeführt. In der *strukturellen Phase* wird die Struktur des Tests bzw. Fragebogens mittels geeigneter teststatistischer Verfahren und Kennwerte überprüft. In einer letzten Phase (*externe Phase*) werden weitere Belege für die Validität von Test- bzw. Fragebogenergebnissen gesammelt. Die Validierung solcher Verfahren stellt jedoch keinen abschließenden Schritt der Test- bzw. Fragebogenkonstruktion dar, denn sie wird in allen Phasen der Test- bzw. Fragebogenkonstruktion berücksichtigt. Die Validierung von Test- und Fragebogenverfahren ist theoretisch und methodisch anspruchsvoll und sollte nie als abgeschlossen betrachtet werden. Es sollte strenggenommen auch nicht davon gesprochen werden, dass ein Test bzw. Fragebogen valide ist, da Validität keine Eigenschaft solcher Verfahren ist. Es können nur Aussagen und Schlussfolgerungen valide sein, die auf der Grundlage von Test- bzw. Fragebogenergebnissen getroffen werden.

## 1. Goal of this how-to article

In *medical education* research, tests and questionnaires are often used, for example, to measure motivation, empathy, or certain performance levels of students. However, the training of physicians and medical professionals hardly conveys the competencies that would enable them to evaluate the quality of such measures, develop measurement instruments, or translate an existing questionnaire. This *how-to article* is intended to explain and illustrate the development of psychological test and questionnaire measures.

The process of test or questionnaire construction is complex and time-consuming. There are various specialised books in which this process is described in detail, usually on more than 200 pages [1], [2]. A short article can therefore only enable readers to roughly evaluate tests and questionnaires and convey an idea of how such measures are generally constructed according to the so-called *Classical Test Theory* (CTT) (see below).

The following paragraphs will first briefly explain the different types of tests and questionnaires. Then, the various phases of constructing such measures will be outlined.

## 2. What types of test or questionnaire measures can be distinguished?

*Psychological test* and *questionnaire measures* can be assigned to three areas: performance tests, personality questionnaires, and projective techniques [3]. Each area can be further subdivided (see table 1 (Tab. 1)). These measures may cover abilities, skills, characteristics, and states of persons that often are not directly observable, but derived from observable behaviours, and referred to as constructs. Well-known psychological constructs used in *medical education research* are, for example, motivation, self-efficacy, resilience, reflective ability, and empathy. Since constructs cannot be directly measured, they are referred to as latent variables, for which items are used as indicators [2].

Most psychological test and questionnaire measures are based on the assumptions and construction principles of the so-called *Classical Test Theory* (CTT) [4], [5], which assumes that individual measurements can vary across different points of measurement. Its basic concept involves the assumption that the observed value X of a person on a test consists of both the person’s *true score* and a *random measurement error*. The result of an intelligence test would accordingly be influenced by the actual intelligence of the person being tested and by unsystematic influences, such as performance fluctuations due to the time of day, e.g. if one were to conduct an infinite number of measurements, the mean of these measurements would correspond to the person’s actual intelligence score.

In addition to the CTT, there is the *Probabilistic Test Theory* (PTT), which is sometimes also called *Item-Response Theory* (IRT). This theory assumes that the probability of a specific response to an item depends on the characteristics of the item and the level of the latent trait being measured in the person [5]. According to Rost [4], the two test theories CTT and PTT are not, as often described, competing, but complementary methods, since one theory starts where the other ends, or because both test theories are largely based on the same assumptions. Further details on PTT can be found in Bühner [2] and Döring and Bortz [5].

## 3. How are test and questionnaire measures developed?

When developing a test or a questionnaire measure, so-called test quality criteria must be fulfilled (see table 2 (Tab. 2)). The development of such measures begins with the determination or definition of the construct to be measured. After that, items (tasks or statements) are constructed, and the answer format is selected. After a pretest, the measure is specifically tested on one or more samples. If a sufficiently large number of data has been obtained, it is analysed how reliable the test or questionnaire measures the construct (reliability) and whether it measures the construct it claims to measure (validity).

### 3.1. Definition of the construct

To define and operationalize the construct, theories or models are used, if available. Examples of constructs based on sound theories and models that have been used to develop psychological tests include motivation and learning strategies. If theories and models are not available, the construct space can be narrowed down after extensive literature study, and indicators of the construct (e.g. specific statements or behaviours) can be determined. A current example from medical education research where such a procedure is necessary is* reflective ability*. There are various models and theories here as well, but they differ significantly in what is understood by *reflective ability*. Therefore, to develop a test procedure for *reflective ability*, it would first be necessary to define which indicators of *reflective ability* should be considered based on prior work. As part of the construct definition, it should also be determined to what extent relationships and overlaps with other constructs exist (nomological network) [2]. For example, there has been an illustrative discussion as to the extent to which the personality trait of *openness to experience* is related to creativity [6].

The quality of the definition of the construct determines how easily items can be generated. A detailed definition considering necessary distinctions from other constructs also increases the likelihood of the *content validity* of the construct [1], [2].

### 3.2. Generation and wording of items

Different sources can be used to generate items [1]. For example, items can be

- derived from *theories* or from an extensive, systematic review of the *literature,*
- generated from the results of *preliminary investigations* (interviews, focus group discussions, etc.),
- written in accordance with *existing tests and questionnaires,*
- developed by *experts*.

When generating items, the goals of the test being constructed should be considered [2]. If the goal is to capture the trait or ability manifestations of individuals, content-valid items should be constructed. A test for detecting *fear of progression*, i.e. the fear a diagnosed condition might progress and deteriorate, is valid in terms of content if the test items can be considered a representative sample of the entire range of *fear of progression* (e.g. cognitive, emotional, and behavioural aspects). It should be ensured that only one construct is captured with the items. Furthermore, all indicators of a construct should correlate with each other [2].

To ensure the *content validity* of the test, attention should be paid to collect a sufficiently large and representative number of items. The number of items in the drafted test should be greater than the planned number of items in the final version [2].

Before constructing the items, it should be decided how exactly items should be written. For example, this can be done in the following ways:

- As *questions*: Do you feel respected by members of other health professions?
- As *statements*: I feel respected by members of other health professions.
- In the *first person singular*: I enjoy working with members of other health professions.
- In an *impersonal form*: People enjoy working with members of other health professions here.

The items should be coherent and understandable in terms of content [1], [2]. This means, among other things, that foreign words or complex sentence structures should be avoided. The items should also be clearly defined in terms of content. For this purpose, if possible, avoid conditional statements or conjunctions, among other things. Negations (especially double negatives) should also be avoided.

### 3.3. Choosing the response format

The selection of appropriate response options is just as important as constructing the items. Frequently, psychological test and questionnaire measures use rating scales (usually so-called Likert scales), with graded response categories to which verbal labels are attached. Labels often encountered range from “not applicable” to “applicable” or “very poor” to “very good”. Rating scales may also differ in the number of response categories. In this regard, response scales with up to 7 levels are acceptable [2]. Furthermore, it must be decided whether the response levels of the items are unipolar (e.g. “never” to “very often”) or bipolar (e.g. “disagree”, “slightly disagree”, “neither disagree/nor agree”, “slightly agree”, “agree”). In addition to verbal labels of the response levels, visual aids can also be used (e.g. smileys).

### 3.4. Wording of the instruction

The purpose of the instruction is to familiarize respondents with the content and purpose of the test or questionnaire measure, provide guidance on how to answer the items, and explain data protection regulations [7]. It has a central function, as it not only prepares for the task of taking the test, but can also create a pre-set attitude in the people being questioned about the task to be completed [1]. An instruction is usually drafted at the end of the construction process, after the items and response alternatives have been determined. In addition to specifying the objective or purpose of the test or questionnaire, instructions usually contain information indicating that

- participation is voluntary and that there are no disadvantages to be feared in case of non-participation,
- all items should be read and answered quickly,
- the items are to be responded to one after the other and no item should be skipped, even if this may seem difficult at times, and that in this case the “most likely” option should always be checked,
- confidentiality and anonymity of individual information is ensured in accordance with applicable data protection regulations.

### 3.5. Conducting preliminary tests

Conducting one or more pretests is another important prerequisite for the development of a test or questionnaire measure. However, there are no generally accepted procedural rules for carrying these out. For example, recommendations vary greatly when it comes to determining the number of cases necessary for this [8]. However, a small number of individuals are usually asked to provide feedback on the comprehensibility of the items and instructions, and to report any difficulties encountered while completing the measure. It is important that these individuals are as similar as possible to the subsequent target group of the test or questionnaire, e.g. in terms of language comprehension. Preliminary tests also provide information about the time needed for completion, the respondents’ interest in the topic, and the possible distributions of the responses. Based on the feedback, the measure will be modified if necessary.

## 4. Statistical evaluation of psychological test and questionnaire measures

The process of statistically evaluating a test or questionnaire measure can be subdivided in accordance with phases outlined by Loevinger [9], as follows:

- *Substantive phase*: During this phase, the measure is theoretically grounded and based on available literature. Pretests are conducted to clarify the comprehensibility of the items and problems with answering them.
- *Structural phase*: The primary focus of this second phase is on examining the structural (e.g. factorial structure) and further psychometric properties (e.g. item correlations) of the measure.
- *External phase*: In this phase, the extent of the agreement of the measure with other criteria and, if applicable, similar tests or questionnaires should be determined.

All previous descriptions in this *how-to article* can be assigned to the *substantive phase* (see table 3 (Tab. 3)). The following sections focus on the psychometric analysis of test or questionnaire measures that are assigned to the other two phases.

### 4.1. Structural phase

In the *substantive phase*, the *face* and *content validity* of test or questionnaire measures can already be ensured. However, the structural and psychometric properties of test or questionnaire measures can only be determined after the test and questionnaire measure has been taken by individuals from the respective target group (data collection). First, a dimensional analysis should be performed using factor analyses (statistical methods that group the variables according to their intercorrelation; *factorial validity*), followed by determining the test’s reliability and an item analysis [10]. However, if the sample size is too small [2] for dimensional analyses, preliminary reliability calculations can be conducted and the items can be analysed regarding their difficulty, discriminant validity, and intercorrelations (item analyses) (see table 4 (Tab. 4)). 

Recommendations for the sample size required for factor analyses vary greatly in the relevant literature. According to MacCallum et al. [11], common rules of thumb are problematic because the required sample size depends on the number of items per factor and the degree of communality (the proportion of variance of a variable that is explained by the factors) of each item. However, communalities are usually not known in advance. Therefore, in spite of the aforementioned issues, it may be mentioned here for rough orientation that it has been recommended to include a number of respondents in factor analyses that is at least five to ten times the number of items.

If the sample size is sufficient for conducting factor analyses and a hypothesis or model for the dimensions of the test is available, a *confirmatory factor analysis* should be conducted. If there are no reasonable assumptions about the relationships between the items, an *exploratory factor analysis* is recommended.

### 4.2. External phase

The validation of test and questionnaire measures is theoretically and methodically demanding and should never be considered complete [5], [12]. Therefore, strictly speaking, it should not be said that a test or questionnaire is valid, since validity is not a property of tests or questionnaires (see 4.2.2). Only statements and conclusions based on test or questionnaire scores can be more or less valid. 

The validation of test and questionnaire measures (or more precisely, of test or questionnaire scores) involves a variety of aspects. In this regard, however, the understanding of which indicators can be considered as signs of validity has changed over time. The traditional concept of validity is presented first, followed by the validity approach of Messick [13], which complements the traditional approach.

#### 4.2.1. Construct and criterion validity

First, it can be determined whether the construct captured by the test or questionnaire measure correlates with other theoretical constructs in terms of content and theory (*construct validity*) and/or whether the test or questionnaire scores correlate positively with behavioural manifestations outside of the testing situation (*criterion validity*) [5].

To determine *construct validity,* additional measurement instruments can be used that capture either construct-related or construct-unrelated characteristics. According to Campbell and Fiske [14], in the first case *convergent validity* would be checked and *discriminant validity* in the second. *Construct validity* also includes the previously described *factorial validity* (see 4.1). Furthermore, it is possible to analyse differences in the test results of selected groups. That is, differences in test scores of various groups (e.g. differing by age, socioeconomic status, or education) are postulated based on theoretical considerations and empirical findings [10]. If these differences are found as predicted, they will be interpreted as evidence of validity.

In terms of *criterion validity*, several types of validity can be distinguished depending on the time of measurement of the external criterion [5]. *Retrospective validity* is checked when a criterion (e.g. past school grades) has been collected before the test scores to be validated (e.g. school performance test) is applied. In* concurrent validity*, the criteria (e.g. complaints in medical consultations such as sleeplessness and listlessness) are recorded (almost) at the same measurement time as the test scores to be validated (e.g. results of a measuring instrument for recording the extent of depression). In *predictive validity* the criterion score (e.g. academic performance) is recorded later than the test score to be validated (e.g. results of a medical college admission test). Determining *criterion validity* requires that the chosen external criterion is reliable and valid.

*Incremental validity* is also a type of *criterion validity*, but it is rarely tested. If *incremental validity* is analysed, an established test or questionnaire measure is used that claims to measure the same characteristic as the measure to be validated. The new measure should then significantly improve the prediction of the external criterion [5].

#### 4.2.2. Argument-based validation concepts

The classical concept of validity described in the previous section was expanded by Messick [13]. He describes six general validity aspects, which apply to all diagnostic measurements in the educational sector. They are based on the fundamental idea that the validity of a diagnostic measurement cannot be considered solely as a numerical coefficient, but rather as a theoretically and empirically founded argument for the validity of test score interpretations. In other words, “it is incorrect to use the unqualified phrase* the validity of the test”* ([15], p.11), because the observed test scores are not only a function of the items but also depend on the respondents and the context of the evaluation [13]. Validity can therefore be understood as an argument for the validity of the interpretation of test scores based on evidence regarding these six aspects. In table 5 (Tab. 5), the validity aspects described by Messick are presented. It becomes clear that only the aspects of *substantive validity,* *generalizability* and of *consequential validity* supplement the traditional approach (see table 5 (Tab. 5)).

Additionally, Messick [13] pointed out two potentially confounding variables that could affect validity. A construct may be *underrepresented* because it is too narrow and does not cover important dimensions or facets of the construct. This would be the case, for example, if a test of performance anxiety only captures its emotional component and disregards its cognitive and physiological components. However, validity can also be limited by *construct-irrelevant variance*, if test items are too difficult or too easy for some individuals [13]. This is the case, for example, when the correct completion of tasks in a mathematics test also depends on its unreasonably high demands on the respondents’ language comprehension.

These expansions of the classical concept of validity have by now been adopted by, among others, the *American Educational Research Association* (AERA) and the *American Psychological Association* (APA) [15], [16].

## 5. Translation of a test or questionnaire measure

In the past, tests were often translated using the forward-backward-translation method. That is, the test was first translated into the target language, then this translation was re-translated [17] by another person, and then the original and the backward-translated versions were compared. However, a simple backward translation cannot eliminate all translation problems, so multi-stage translation processes are now recommended [17]. For example, according to the *European Social Survey Programme* for translating questionnaires, a five-step translation framework called *TRAPD* is suggested. This acronym stands for *T*ranslation, *R*eview, *A*djudication (deciding on a version), *P*re-testing, and *D*ocumentation [18]. These five steps should be taken in a team effort from the beginning. A complete statistical evaluation of the translated version is also required when translating a test.

## 6. Summary

The construction of test or questionnaire measures requires a well-defined construct or at least a clearly described construct space. Based on this, items can be written that must be content-valid and easy to understand and that are oriented toward the goals of the measure. If the measure has been supported in pretests with small groups of people, its structural (dimensionality) and further psychometric (reliability, validity, etc.) properties can be checked using more extensive data collections. To determine the validity of the test results, various aspects need to be considered. These relate primarily to the construct to be measured and its theoretical embedding as well as to its relationship to other variables, but also to the context of the measurement and the consequences derived from the test results.

## Authors’ ORCIDs

- Marianne Giesler: [0000-0001-9384-2343]
- Götz Fabry: [0000-0002-5393-606X]

## Competing interests

The authors declare that they have no competing interests. 

## Figures and Tables

**Table 1:**
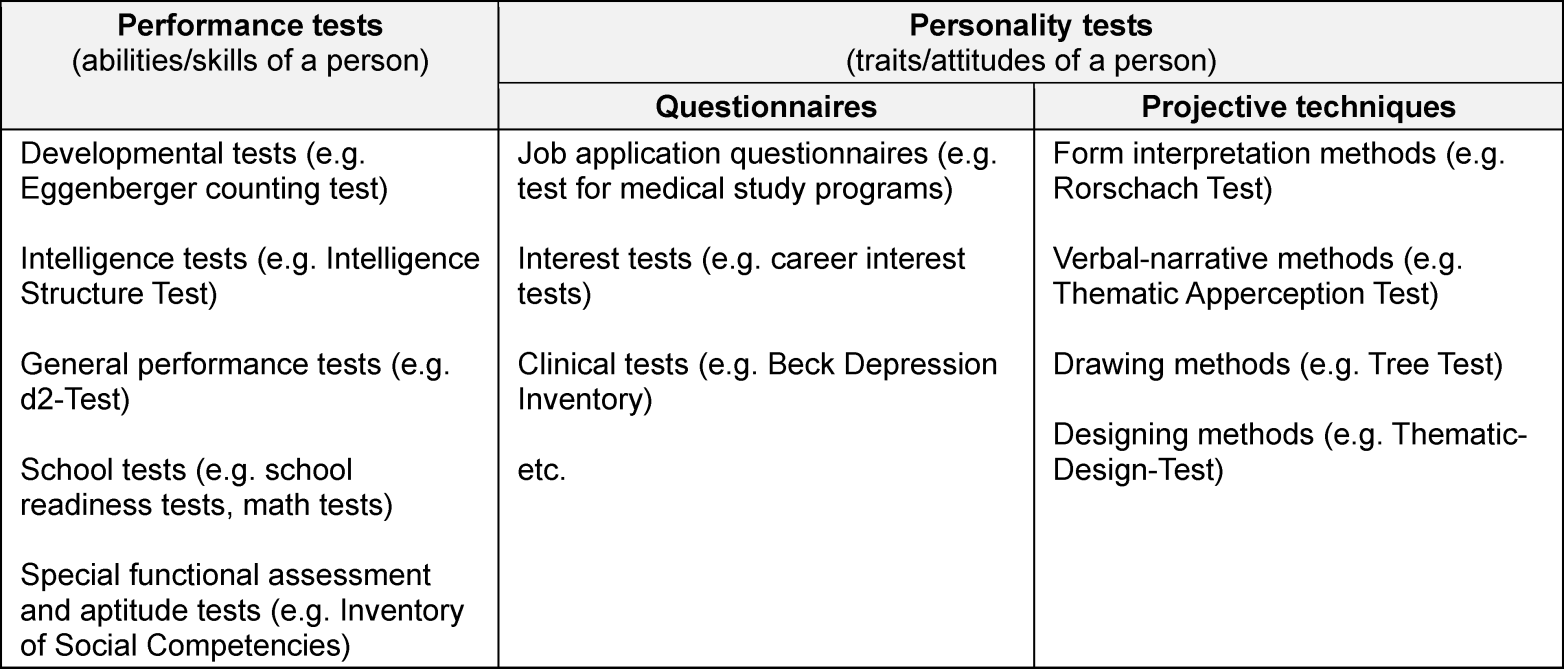
Psychological test and questionnaire measures – an overview with examples [3]

**Table 2:**
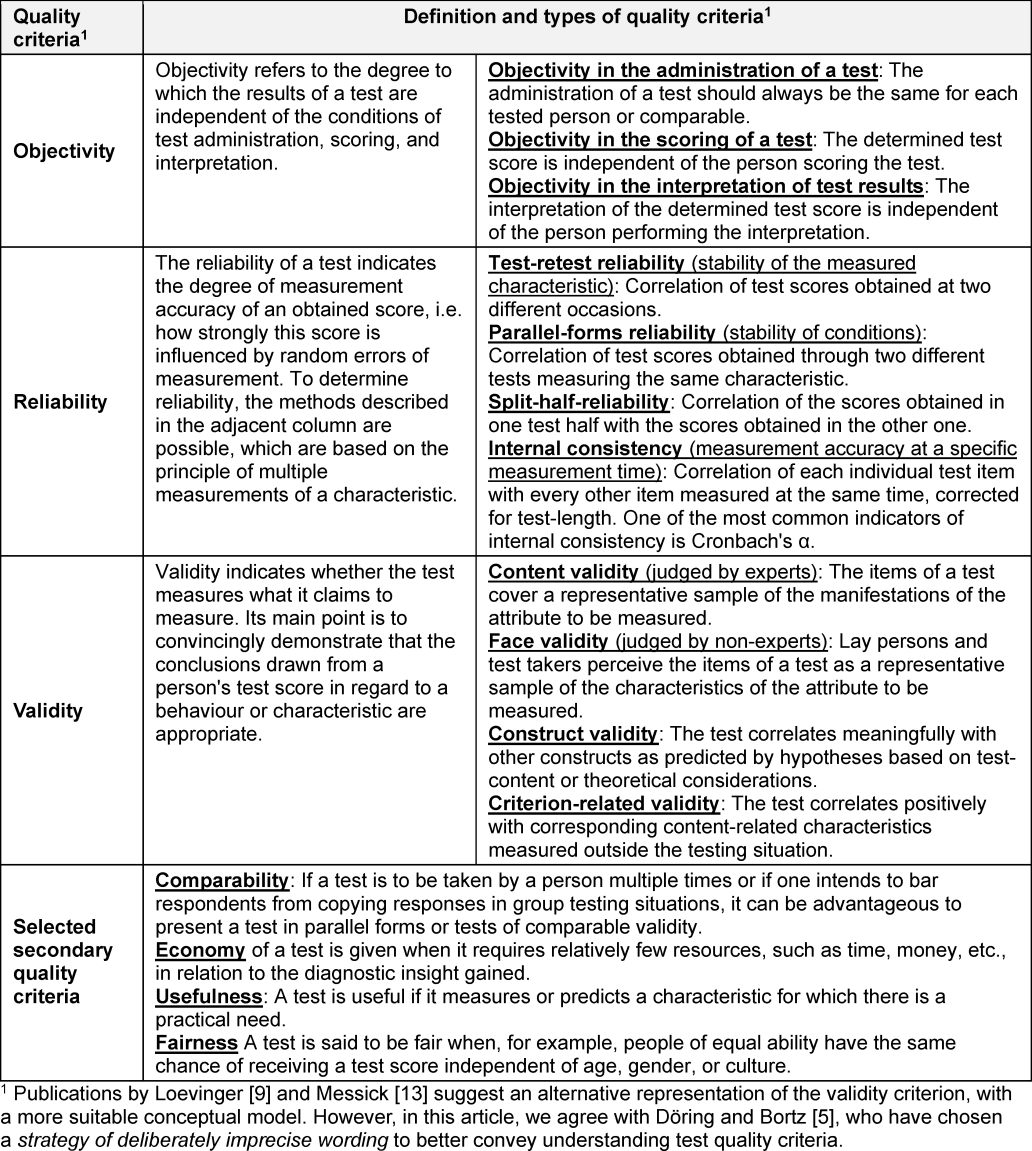
Traditional quality criteria of tests [2], [5], [10], [19]

**Table 3:**
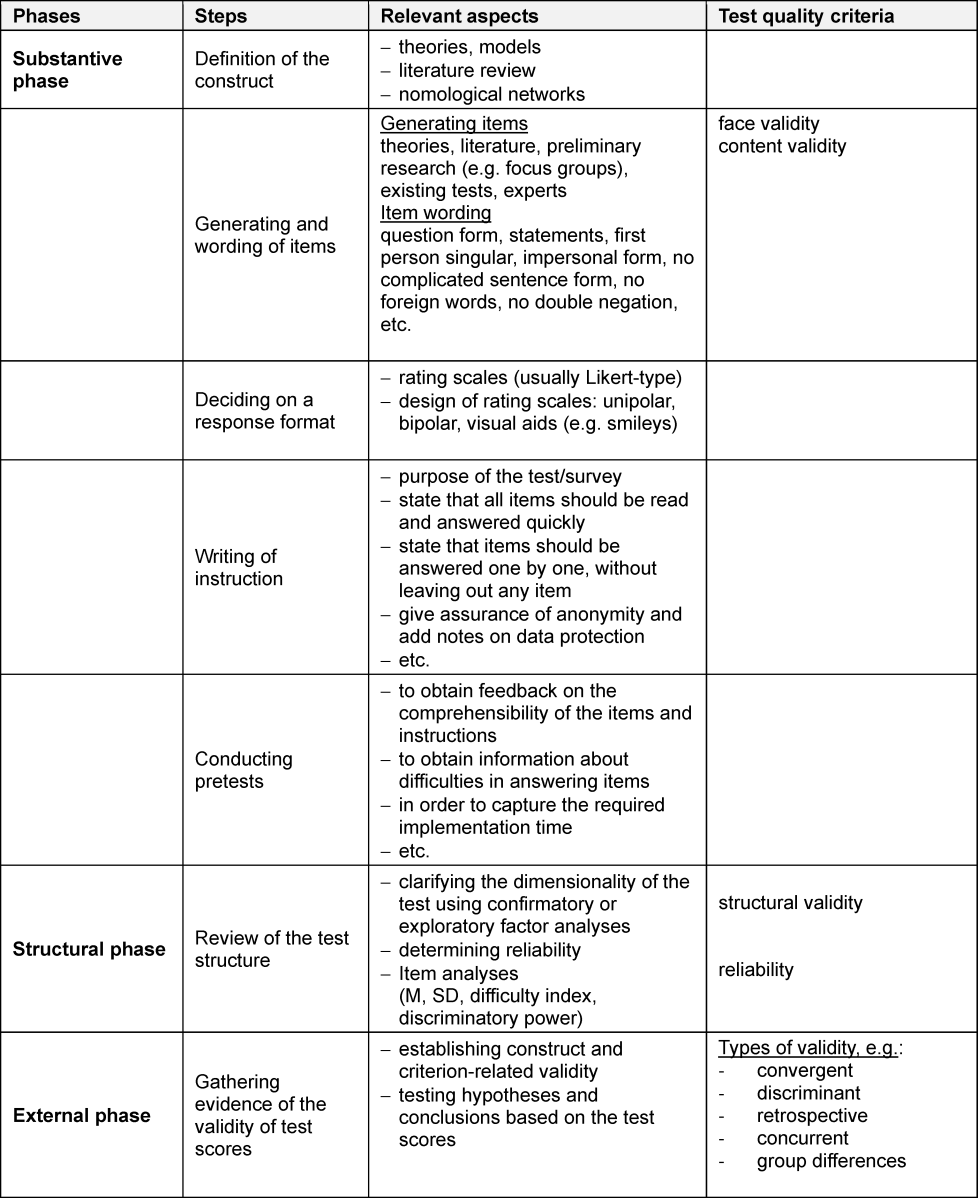
Phases of constructing test and questionnaire measures (see chapter 4)

**Table 4:**
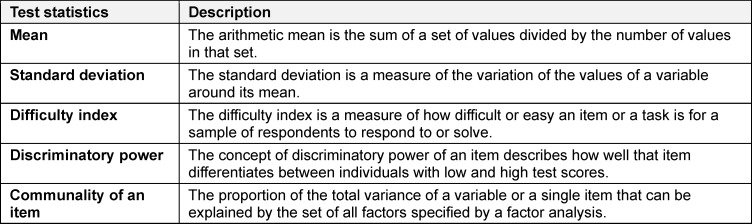
Description of test statistics

**Table 5:**
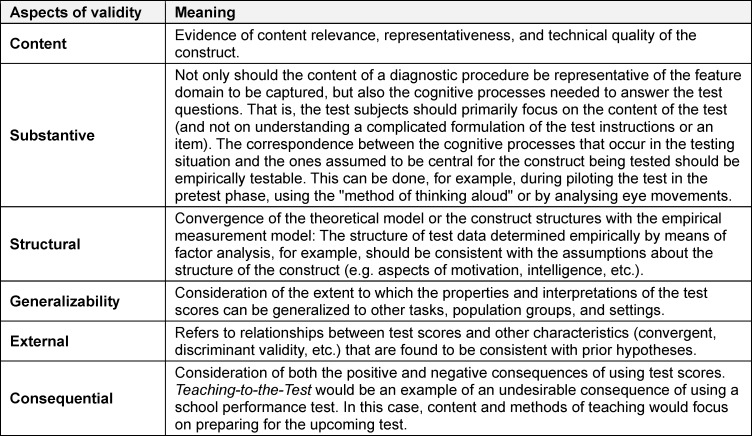
Validity approach by Messick [5], [13], [20]
